# Using 164 Million Google Street View Images to Derive Built Environment Predictors of COVID-19 Cases

**DOI:** 10.3390/ijerph17176359

**Published:** 2020-09-01

**Authors:** Quynh C. Nguyen, Yuru Huang, Abhinav Kumar, Haoshu Duan, Jessica M. Keralis, Pallavi Dwivedi, Hsien-Wen Meng, Kimberly D. Brunisholz, Jonathan Jay, Mehran Javanmardi, Tolga Tasdizen

**Affiliations:** 1 Department of Epidemiology and Biostatistics, University of Maryland School of Public Health, College Park, MD 20742, USA; yorohuang@gmail.com (Y.H.); jkeralis@umd.edu (J.M.K.); dwvdpallavi@gmail.com (P.D.); sherrytpe@gmail.com (H.-W.M.); 2 School of Computing, Scientific Computing and Imaging Institute, University of Utah, Salt Lake City, UT 84112, USA; abhinav3663@gmail.com; 3 Department of Sociology, University of Maryland, College Park, MD 20742, USA; hduan1@umd.edu; 4 Intermountain Healthcare Delivery Institute, Intermountain Healthcare, Murray, UT 84107, USA; Kim.Brunisholz@imail.org; 5 Department of Community Health Sciences, Boston University School of Public Health, Boston, MA 02118, USA; jonjay@bu.edu; 6 Department of Electrical and Computer Engineering, Scientific Computing and Imaging Institute, University of Utah, Salt Lake City, UT 84112, USA; mehjavan@gmail.com (M.J.); tolga@sci.utah.edu (T.T.)

**Keywords:** COVID-19, built environment, big data, GIS, computer vision, machine learning

## Abstract

The spread of COVID-19 is not evenly distributed. Neighborhood environments may structure risks and resources that produce COVID-19 disparities. Neighborhood built environments that allow greater flow of people into an area or impede social distancing practices may increase residents’ risk for contracting the virus. We leveraged Google Street View (GSV) images and computer vision to detect built environment features (presence of a crosswalk, non-single family home, single-lane roads, dilapidated building and visible wires). We utilized Poisson regression models to determine associations of built environment characteristics with COVID-19 cases. Indicators of mixed land use (non-single family home), walkability (sidewalks), and physical disorder (dilapidated buildings and visible wires) were connected with higher COVID-19 cases. Indicators of lower urban development (single lane roads and green streets) were connected with fewer COVID-19 cases. Percent black and percent with less than a high school education were associated with more COVID-19 cases. Our findings suggest that built environment characteristics can help characterize community-level COVID-19 risk. Sociodemographic disparities also highlight differential COVID-19 risk across groups of people. Computer vision and big data image sources make national studies of built environment effects on COVID-19 risk possible, to inform local area decision-making.

## 1. Introduction

The COVID-19 pandemic has caused approximately 150,000 deaths in the United States as of 29 July 2020 [1], and has had unprecedented negative effects on the U.S. economy and households in numerous ways. The unemployment rate rose up to 14.9% in April and the GDP fell by 1.2% in the first quarter in 2020, which is the largest decline since the Great Recession [2,3]. Yet the negative impacts of COVID-19 are not evenly distributed. About half of lower-income U.S. households lost employment income. About 62% of Hispanics and 57% of Black adults were in households that experienced employment income loss compared to 45% of whites [4]. Moreover, the spread of COVID-19 is not evenly distributed. Racial/ethnic disparities in COVID-19 infection and mortality are coming to light, with disproportionate numbers of COVID-19 cases and deaths among racial/ethnic minorities compared to non-Hispanic whites [5,6]. Some of these differences reflect the living and social conditions faced by racial/ethnic minorities. For instance, institutional racism that produced residential segregation may increase the likelihood that racial/ethnic minorities live in densely populated areas with substandard and crowded housing conditions impede social distancing [7,8]. A recent analysis suggested that counties that are predominately black have three times the infection rate of COVID-19 compared to white majority counties [9,10].

COVID-19 can spread through droplets that are released when people talk, cough or sneeze or when people touch a contaminated surface and then touch their nose or mouth [11]. Research has identified a myriad of important factors that influence COVID-19 transmission including anti-contagion governmental policies [12], community adherence to preventative health behaviors (e.g., mask wearing, social distancing) [13] and other environment characteristics like air pollution. Emerging research has found higher levels of air pollution may increase COVID infection rates as well as COVID-related mortality, possibly because particulate matter can act as a carrier of the virus and also compromise the baseline health of communities that have chronic exposure to air pollution [14]. In the current study, we focus on a neglected area of research, the potential relationship between built environment characteristics and COVID-19 cases. To conduct this investigation, we utilized the largest collection of Google Street View images that has been leveraged for public health research to characterize neighborhood environments. In examining associations between built environment characteristics and COVID cases, we controlled for demographic compositional characteristics of areas and population density, which has previously been utilized in econometric studies as a proxy for air pollution and other factors found with greater prevalence in urban areas [15,16].

Neighborhood environments may structure risks and resources [17] that produce COVID-19 disparities through several pathways. Firstly, neighborhood built environments that allow greater flow of people into an area or impede social distancing practices may increase residents’ risk for contracting the virus. A recent study that used data from pregnant women in New York City revealed that overcrowding housing units have higher chances of contracting COVID-19 [18]. Neighborhoods with a mixture of residential and commercial uses (e.g., high prevalence of grocery stores and businesses), multiple lanes of traffic, and higher density of sidewalks, may allow more people to congregate in an area and more easily spread COVID-19.

Additionally, previous studies found that physical disorder in the neighborhood environments is significantly associated with higher prevalence of chronic diseases [19] and poor self-rated health [20], which also increases the chances of contracting COVID-19 [21,22]. Physical disorder refers to features of the environment that signal decay, disrepair, and uncleanliness. Examples of neighborhood indicators of physical disorder include vacant or abandoned housing, vandalized and run-down buildings, abandoned cars, graffiti, and litter [23]. Physical disorder is often interpreted as an indicator of low neighborhood quality [24]. Physical disorder is hypothesized to indicate a breakdown of social disorder and control, which reduces individual well-being and increases fear, mistrust, isolation, anger, anxiety, and demoralization [25]. Mechanisms proposed include the daily stress imposed by environments that are deemed unsafe. Previous research has connected physical disorder with an array of detrimental health outcomes including worse mental health, higher substance use, physical functioning and chronic conditions [26]. Physical disorder might also indicate fewer resources for infrastructure maintenance and investment. Communities with poor-quality housing stock may have less healthy indoor conditions, with consequences for baseline respiratory health.

In this study, dilapidated buildings and visible utility wires overhead were utilized as indicators of disorder. Visible utility wires hanging overhead are visually striking and may impact residents’ aesthetic sense of their environment, altering perceptions of safety or pleasurability and influencing both mental health (by affecting stress levels) and physical health (by disincentivizing walking). Other studies that have examined this indicator have been done outside the U.S., where they may also represent an unsightly presence and electrocution/electrical fire risk [27]. Computer vision models have struggled with small objects, precluding us from labeling other indicators of physical disorder such as litter or trash [19].

Investigations into neighborhood conditions are typically conducted on small scales for only certain cities or neighborhoods [28,29]. When conducted, neighborhood data collection is expensive and time consuming, and then only available for certain time periods. Currently, detailed neighborhood data come from neighborhood surveys, administrative data such as census data, and systematic inventories of neighborhood features. Subjective assessments of neighborhoods from community residents can help identify factors that residents believe are most important to their health and increase understanding on how individuals differentially use and interact with their environment. However, self-reported neighborhood data can be influenced by participants’ health status and cognitive function, resulting in “single source bias” [30]. The other neighborhood data we do have is mainly data on demographics (e.g., percent black). To our knowledge, our study is the largest to date using zip code level cases from 20 states to investigate associations between built environments and COVID-19 cases. Previous studies examining the distribution of COVID-19 cases are only focused on one or two states [31,32,33] or larger geographies like counties [34].

Google Street View (GSV) images represent a massive, publicly available data resource that has high potential but is very underutilized for health research. It can be used to extract information on physical features of the environment at point locations all over the country. Consistently constructed neighborhood quality indicators across large areas are severely lacking. While some studies have used human coders to classify environmental features seen in Google Street View images [35] this approach is not feasible on the massive scale necessary to compare thousands of U.S. neighborhoods. The development of data algorithms that can automatically analyze big data sources such as street view images will create a new national data resource for timely decision-making to mitigate the impact of COVID-19 and future outbreaks on health and health disparities. The purpose of characterizing built environments that have higher COVID-19 risk is to identify places where additional safeguards and resources are needed.

Study aims and hypotheses. In this study, we investigated how the built environments affect COVID-19 cases at the zip code level. We utilized 170 million GSV images sampled at 50 meters apart and computer vision models to comprehensively characterize neighborhood conditions across the United States. From GSV images, we created indicators of urban development (non-single family home, single lane roads), walkability (crosswalks, sidewalks), and physical disorder (dilapidated building, visible utility wires). We hypothesize that built environments characterized by greater urban development, walkability, and physical disorder will have higher COVID-19 infection rate.

## 2. Materials and Methods

Street View image data collection. We utilized Google Street View’s Application Programming Interface (API) to capture street view images of our search set. Image resolution was 640 × 640 pixels. We surveyed all U.S. roads and obtained 4 images from each sample location with angle views at 0, 90, 180, and 270 degrees, thus permitting fuller capture of the surrounding area of a point location. In total, 164 million images were obtained in November 2019.

Image data processing. Convolutional Neural Networks (ConvNets) [36,37,38] achieve state-of-the-art accuracy for several computer vision tasks including but not limited to object recognition, object detection, and scene labeling. For example, the state-of-the-art accuracy of ImageNet [39] with 1000 categories and over one million image samples is improved every year using ConvNet-based methods. The ImageNet dataset contains images from various categories (e.g., “moped”, “Granny Smith apple”) and corresponding category labels. Models trained on this dataset use trial and error to learn combinations of colors, shapes, and textures that are relevant to a wide variety of image interpretation tasks, and therefore can be used as a starting point for creating computer vision models for tasks where labeled training data is scarce. A ConvNet model “pre-trained” on ImageNet can be “fine-tuned” using a smaller amount of training data from the desired task, which delivers strong classification performance without requiring the vast training data and computational resources necessary to train the original ConvNet.

Neighborhood definitions. Zip codes were utilized as neighborhood boundaries because various health departments across the country are releasing COVID-19 cases by zip code. To arrive at the neighborhood indicators, we processed street imagery and then combined information on all street imagery within a zip code to arrive at zip code-level summaries (e.g., the percentage of images in a zip code that contain a sidewalk).

Built environment indicators. To create a training dataset for our computer vision models, from December 2016–February 2017, we manually annotated 18,700 images (from Chicago, Illinois; Salt Lake City, UT; Charleston, West Virginia; and a national sample). These locations were chosen to capture heterogeneity in neighborhood environments across geographically and visually distinct places with varying population densities, urban development, and demographics. Labelers included the principal investigator and three graduate research assistants. Inter-rater agreement was above 85% for all neighborhood indicators. Each image received labels for these binary neighborhood characteristics: (1) street greenness (trees and landscaping comprised at least 30% of the image—yes/no), (2) presence of a crosswalk, (3) single lane road, (4) building type (single-family detached house vs. other), and (5) visible utility wires. Green streets were utilized to indicate lower urban development. Single lane/residential roads limit the number of cars and hence flow of people. Non-single family home was utilized as an indicator of residential and commercial mixture. Crosswalks were utilized as an indicator of walkability. Visible utility wires were utilized as indicators of physical disorder.

We randomly divided the dataset into a training set, a validation set, and a test set. The training and validation set contained 80% of total labeled images and the remaining 20% was used as a test set to evaluate the model’s performance. Once the hyper-parameters were chosen, each model architecture was trained multiple times. Note that neural network training is stochastic even when starting from the same initialization and using the same training set, therefore, multiple training runs are used to assess the mean and standard deviation of the error. The testing set remained unobserved until the best models had been selected using the training set. We assessed the final quality of the model using the test set. We first resized all the images to the size 224 × 224 for processing. We then trained a standard deep convolutional neural network architecture—Visual Geometry Group VGG-19 [36] in Tensorflow [40] with sigmoid cross entropy with logits as the loss function. The weights of the network were initialized from ImageNet weights. Adam optimizer was used with batch size 20. Training took 20 epochs and started with learning rate 10^−4^. We considered the model saved in the last epoch as our final model. Accuracy of the recognition tasks (agreement between manually labeled images and computer vision predictions) were the following: street greenness (88.70%), presence of crosswalks (97.20%), non-single family home (82.35%), single lane roads (88.41%), and visible utility wires (83.00%). These figures were consistent with a separate, semi-supervised learning approach. Below, we describe the model building process for two additional neighborhood indicators that utilized different training datasets.

Dilapidated building indicator. Our training dataset consists of approximately 29,400 Google Street View images captured from Baltimore and Detroit based upon administrative lists from city governments on vacant buildings and buildings marked for demolition from 2014–2018. We randomly split this dataset in the ratio 80:20 for validation to obtain about 23,500 images for training and 5900 for validation. The dataset has an equal number of normal and dilapidated buildings. We then trained a standard deep convolutional neural network architecture- ResNet-18 [38] in Pytorch [41] with NLL loss as the loss function. For the dilapidated building indicator, the ResNet-18 model produced an accuracy of 89.1% and a F1 score of 89.1.

Sidewalk indicator. Our training dataset consists of about 24,316 images captured from Google Street View from New Jersey that had been manually labeled. We randomly split this dataset in the ratio 80:20 for validation to obtain 19,452 images for training and 4864 for validation. The minority label images were oversampled so that the dataset has an equal number of sidewalk present and absent cases. We then trained a standard deep convolutional neural network architecture—ResNet-18 [38] in Pytorch [41] with NLL loss as the loss function. For the sidewalk indicator, the ResNet-18 model produced an accuracy of 84.5% and a F1 score of 81.0.

COVID-19 cases. To our knowledge, there is no national data source for zip code COVID-19 cases, with the Centers of Disease Control and Prevention and John Hopkins COVID-19 Map only showing county level cases as the lowest level of geography. To obtain zip code COVID-19 cases, we visited state and county health departments that had COVID-19 information (31 websites in total; 12 websites utilize ArcGIS dashboards, and 19 utilized a mixture of pdfs, csv files, and Tableau/PowerBI embedded websites). Data were obtained from official government websites and actively maintained GitHub repositories using various methods. This collection process was automated using Python packages including scrapy, selenium, beautifulsoup, and requests. Specifically, for websites with ArcGIS map layer, we used ArcGIS query services to query the feature layer; for websites with CSV data files to download, we automated the download process from the websites; for static website tables, we leveraged scrapy or beautifulsoup packages to harvest the web content; for websites with PDF files, we first downloaded the PDF files and utilized OCR technology to convert the data into the CSV format. Some states have report data for all zip codes, but others only report for certain cities or counties. Zip code confirmed COVID-19 cumulative cases as of 21 June 2020, were obtained for Arizona, California (Sacramento County, San Francisco County, San Diego County), Colorado (Weld county), Georgia (Fulton County), Florida, Illinois, Maryland, Michigan (Monroe County, Kent County), Missouri (St. Louis), New Mexico, New York City, North Carolina, Oklahoma, Oregon, Pennsylvania, Rhode Island, Texas (Harris County, Fort Bend County, Travis County, Collin County, Denton county, Tarrant County), Utah (Salt Lake City), Virginia, Washington State (Spokane County). COVID-19 cases varied across zip codes with some zip codes reporting zero or few cases and others reporting hundreds of cases. About 50% of zip codes had 15 or fewer cases (“cold spots”) and 10% had 250 or more cases (“hot spots”). In this study, we investigated whether zip code built environments can help explain some of the variation in COVID-19 cases across 20 states.

### Statistical Analyses

For each zip code, we calculated the percentage of total number of images that contained a given built environment indicator (e.g., number of images with a sidewalk/total number of images) *100 = percent with sidewalk. From there, we created tertiles and classified each zip code based on their percentage, with the lowest tertile as the reference group. We fit Poisson regression models to estimate associations between GSV-derived built environment characteristics and COVID-19 cases, controlling for potential confounding variables. Log of total population at risk was used as the offset variable, to account for varying population sizes across zip codes. Goodness-of-fit chi-square tests indicated the data fit with the Poisson model form. All predictor variables were standardized with a mean of 0 and a standard deviation of 1. Coefficients from Poisson regression models were exponentiated to arrive at estimates of incidence rate ratios for a one-unit change in the predictor variable (i.e., one standard deviation change). Separate regressions were run for each built environment indicator given moderate associations between the built environment indicators that varied from −0.23 for single lane roads and visible wires to −0.83 for green streets and non-single-family homes. Models controlled for population density, household size, median age, household income, poverty rate, unemployment, percent with less than a high school education, percent Asian, percent Black, and percent Hispanic. Covariate information was obtained from the American Community Survey 2018 5-year estimates, with the exception of population density and household size which were obtained from the 2010 US Census.

We hypothesized that zip codes with more crosswalks and sidewalks (indicators of walkability), non-single family homes (an indicator of mixed commercial/residential uses), more visible wires and dilapidated buildings (indicators of physical disorder) would be associated with more COVID-19 cases. We hypothesized that zip codes with more single lane roads (an indicator of lower urban development) would be associated with fewer COVID-19 cases. Stata IC15 (StataCorp LP, College Station, TX, USA) were used for all data analyses. This study was approved by the University of Maryland Institutional Review Board.

## 3. Results

**Figure 1:**
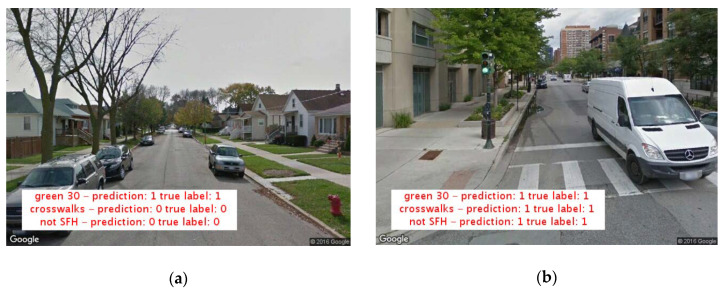
Example processed Google Street View images for green street, presence of crosswalks, and “not single family home” indicators. Predictions were algorithm-derived labels for neighborhood features. “True” labels were manual annotations provided by the research team. (**a**) presents a residential scene with single family homes, ample street landscaping, and no crosswalks present. (**b**) presents a mixed-use neighborhood with ample street landscaping, and a crosswalk present.

Figure 1 presents examples of processed Google Street View images. Predictions were algorithm-derived labels for neighborhood features. “True” labels were manual annotations provided by the research team. Our computer vision model was able to classify even winter scenes as “green streets” because the model was trained with manually annotated images to recognize tree branches as landscaping.

Table 1 displays descriptive statistics at the zip code level. On average, approximately 25% of images in a zip code contained a building that was not a single family home, 20% of images had a sidewalk, 2% with a crosswalk, and 44% with visible utility wires. Dilapidated buildings had a prevalence of 18%, while single lane roads (65%) and green streets were more prominent (87%) (Table 1). We examined COVID-19 cases in 8171 zip codes across 20 states in the United States with an average of around 546 cases per 100,000.

Table 2 presents the results of our Poisson regression analyses examining the relationship between GSV-derived built environment characteristics and COVID-19 cases. We found that zip codes with a standard deviation increase in sidewalks had 40% more cases (Table 2). A standard deviation increase in crosswalks and non-single family homes was associated with 14% and 21% more cases, respectively. We also found that indicators of physical disorder such as dilapidated buildings or visible utility wires were associated with more cases. Alternatively, single lane/residential roads and green streets were associated with fewer cases. Zip codes with a standard deviation increase in single lane roads and green landscaping had 10% and 4% relative fewer COVID-19 cases, respectively.

Additionally, population characteristics associated with increased coronavirus cases included household size, percent with less than a high school education, percent racial/ethnic minorities (in particular percent Black), and population density. Estimates for covariates varied because the GSV-derived variable was different in each of the models. Correlations between covariates and the particular GSV-derived characteristic differed and hence the coefficient estimates for covariates also differed. Nonetheless, the variation in estimates for covariates was generally small/moderate. Across models, a standard deviation increase in percent with less than a high school education was associated with 42–54% increase in COVID-19 cases. Across models, percent black was associated with 17–29% increases in coronavirus cases. A standard deviation in population density was associated with 1–4% more coronavirus cases.

Mobility changes during the COVID-19 pandemic may have increased the importance of neighborhood environments. Google’s community mobility report [42] indicates that out of six categories of movement (retail and recreation, grocery and pharmacy, parks, transit stations, workplaces, and residential), movement volumes declined in all categories except residential and parks (Figure S1). Consequently, the neighborhood environment is crucial for containing the spread of coronavirus, as more residents may have limited activities to their immediate neighborhood surroundings.

## 4. Discussion

Our study finds that neighborhood built environment may influence the spread and containment of COVID-19. Leveraging Google Street View Images, we found that single-lane/residential roads and green streets were associated with fewer cases, while non-single family homes, sidewalks, and physical disorder were associated with more cases in the neighborhood. In other words, COVID-19 risk is highest in more built-up, more walkable, and more physically deteriorated zip codes, and lower in zip codes with smaller, greener streets. These associations persist after controlling for urbanicity and sociodemographic indicators, suggesting a meaningful role for the built environment in influencing COVID-19 risk. The study is one of the first to investigate the effect of neighborhood built environment on the spread of coronavirus at the zip code level.

Single-lane/residential roads and green streets are indicators of lower urban development and lower social contacts. Green streets are especially prevalent in rural areas and suburban areas. Conversely, neighborhood environment indicators such as non-single family homes, sidewalk presence, and physical disorder may facilitate the spread of coronavirus. The ability to perform social distancing is not equally distributed across neighborhoods, and it is more difficult to achieve in highly developed urban areas. One study found that it is impossible to implement effective social distancing in urban areas with homes in close proximity to each other, such as Cape Town [43]. The same argument can also be applied to densely populated areas such as New York City, which was the epicenter of the COVID-19 pandemic in the U.S. Residential settings other than single-family homes—for instance, apartment complexes—are more likely to be the source of infectious disease outbreaks. In 2003, the SARS outbreak started in a 33-floor apartment block in Hong Kong [44]. Shared elevators and shared space are both risk factors for COVID-19 infection. Sidewalks, on the other hand, are likely associated with more walking, and the majority of neighborhood sidewalks do not allow pedestrians to maintain the CDC-recommended 6-foot distance. In this study, we find that indicators of physical disorder (dilapidated buildings and unsightly visible utility wires) were connected with more COVID-19 cases, possibly due to worse health and higher comorbidities that increase in disorderly neighborhoods.

Our study is significant because it strives to identify and make available novel indicators of neighborhood quality by leveraging big data resources and furthering the application of computer vision. We utilized Google Street View images as a time- and cost-efficient data source for the characterization of built environments involving close to 170 million images sampled 50 m apart. The inclusion of 20 different states with varying built environments and COVID-19 burden further strengthened our study. Our study found that neighborhoods with greater urban development, higher walkability, and physical disorder had higher coronavirus cases.

Nonetheless, our study also has limitations. The cross-sectional study design inhibits causal inference. Although we have observed strong associations between neighborhood built environment indicators and coronavirus prevalence, we cannot conclude that these characteristics cause higher COVID-19 rates. Additionally, we were not able to control for local COVID-19 resources (e.g., testing availability). However, we controlled zip code sociodemographic characteristics such as racial/ethnic composition and median income that are correlated with greater resource access. Fine particle air monitor data from the U.S. Environmental Protection Agency (EPA) are not available at the zip code level(https://www.epa.gov/outdoor-air-quality-data/pm25-continuous-monitor-comparability-assessments) and hence, we were unable to account for this characteristic in our analyses. Air pollution can vary between areas and has been related to a variety of acute and chronic conditions [45,46], which can compromise health and place individuals at greater risk for more severe COVID-19 illness. Lastly, the study was U.S.-based; built environments, demographics, health policies, and other considerations vary across international settings and thus our study results might not generalize to other countries. Nonetheless, GSV images have been utilized in international settings to examine neighborhood features, and thus has the potential to enable other countries to examine the influence of built environment characteristics on health and other outcomes [47,48].

Like other modes of data collection, image data can only capture a subset of features of a community. Images do not capture all of the features of the neighborhood environment that may impact health outcomes. For instance, we were unable to capture indicators of perceived safety that impact people’s willingness to walk in an area. Additionally, Google Street View API provides the most recent image available for a location. However, areas differ with regard to the rate at which their GSV image are updated. In our dataset, image dates ranged from 2007–2019 and the median year was 2015. Thus, the neighborhood data for certain areas might not reflect current conditions. Moreover, rural areas tend to have older GSV images than urban areas, which may lead to differential measurement bias. In addition, not all types of built environment characteristics lend themselves to easy extraction by computer vision algorithms. Objects that are small (e.g., litter), vary in appearance (e.g., dilapidated buildings), or are very rare in the dataset (e.g., graffiti) are difficult for computer vision models to predict with high accuracy. Subjective characteristics such as the aesthetics or the visual appeal of an area are also difficult to model with computer vision. For subjective characteristics, use of crowdsource techniques that incorporate ratings from residents and visitors might be an effective way to create area-level ratings that capture the variability in these perceptions. Besides the type of neighborhood features that can easily lend themselves to automatic extraction via computer vision models, the depth of neighborhood features that can be extracted may be limited. Well-known neighborhood audit instruments such as the Irvine-Minnesota Inventory [49] and the Pedestrian Environment Data Scan [50] can involve hundreds of different features. Building a computer vision model to accurately extract each of these hundreds of features would be a difficult task.

Additionally, computer vision models using supervised learning approaches often require large training datasets composed of potentially tens of thousands of manually labeled images to adequately train models and hence investigative teams need to build in time and resources to create these large training datasets. In our study, to create our training dataset, team members took two months to label over 18,000 images for neighborhood characteristics. We also utilized administrative datasets that contained the locations of vacant building and buildings marked for demolition to provide enough training examples for our dilapidated building indicator. The use of computer vision and GSV images enables large studies of neighborhood features across broad geographies. However, the use of these automated technologies might limit the type, variety, and level of detail in neighborhood features that can be examined. For investigators interested in neighborhood characteristics for small areas, manual neighborhood inventories might be the appropriate choice to provide the necessary data. While computer vision is not without its limitations, using computer vision and millions of GSV images was the only feasible way to examine fine area-level built environment characteristics across 20 different states. GSV is a growing new area of research that has immense potential to shed light on the potential influence of neighborhood environments on a variety of health outcomes.

## 5. Conclusions

The contextual factors that influence the spread of the coronavirus risk are poorly understood. With recent advances in computer vision and the emergence of massive sources of image data, we developed a data collection strategy utilizing geographic information systems to assemble a national collection of Google Street View images of all road intersections and street segments in the United States. We utilized this data bank and leveraged computer vision algorithms to produce neighborhood summaries of conditions that are linked with COVID-19 risk through increased opportunity for person-to-person transmission. We found that indicators like greater urban development (mixture of residential and commercial buildings, multiple lanes of traffic), walkability (which may increase contact), and greater physical disorder were related to more coronavirus cases. Our study results can help inform population-based strategies to mitigate COVID-19 risk. A higher level of caution can be recommended for the reopening of communities with a heightened level of risk due to their neighborhood design.

## Figures and Tables

**Table 1:** Descriptive statistics, zip code level.

Characteristic	Number of Images	Number of Zip Codes	Mean (Standard Deviation)
Google Street View			
Non-single family home	164,443,190	30,556	25.62% (21.10)
Sidewalks	164,443,190	30,556	19.50% (24.31)
Crosswalks	164,443,190	30,556	1.56% (3.17)
Visible wires	164,443,190	30,556	44.14% (16.81)
Dilapidated building	164,443,190	30,556	18.04% (11.40)
Single lane road	164,443,190	30,556	65.47% (14.31)
Green street	164,443,190	30,556	87.08% (15.70)
COVID-19 outcomes			
Cases per 100,000		8171	545.86 (1353.86)

**Table 2:** Associations between built environment characteristics and zip code level coronavirus cases, 20 States.

Characteristic	Rate Ratio (95% CI)	Rate Ratio (95% CI)	Rate Ratio (95% CI)	Rate Ratio (95% CI)	Rate Ratio (95% CI)	Rate Ratio (95% CI)	Rate Ratio (95% CI)
GSV indicators							
Non-single family home	1.21 (1.16, 1.25)						
Sidewalks		1.40 (1.34, 1.46)					
Crosswalks			1.14 (1.10, 1.18)				
Visible wires				1.08 (1.03, 1.13)			
Dilapidated building					1.03 (0.99, 1.08)		
Single lane roads						0.90 (0.86, 0.94)	
Green streets							0.96 (0.92, 1.00)
Covariates							
Household size	1.03 (0.99, 1.07)	1.02 (0.99, 1.06)	1.03 (0.99, 1.07)	0.99 (0.95, 1.03)	0.98 (0.94, 1.02)	1.00 (0.96, 1.04)	0.98 (0.94, 1.02)
Median household income	1.17 (1.13, 1.22)	1.12 (1.08, 1.17)	1.15 (1.10, 1.20)	1.18 (1.13, 1.23)	1.17 (1.12, 1.21)	1.16 (1.11, 1.20)	1.17 (1.12, 1.22)
Poverty rate	1.11 (1.05, 1.18)	1.09 (1.03, 1.16)	1.16 (1.09, 1.23)	1.20 (1.13, 1.27)	1.21 (1.14, 1.28)	1.16 (1.09, 1.23)	1.20 (1.13, 1.27)
% Less than H.S. education	1.42 (1.32, 1.52)	1.54 (1.43, 1.65)	1.47 (1.37, 1.57)	1.46 (1.36, 1.57)	1.49 (1.39, 1.61)	1.43 (1.32, 1.53)	1.47 (1.36, 1.58)
Civilian employment	1.07 (0.99, 1.16)	1.12 (1.04, 1.20)	1.07 (0.99, 1.15)	1.05 (0.97, 1.14)	1.05 (0.97, 1.14)	1.03 (0.96, 1.12)	1.05 (0.97, 1.14)
% Asian	1.04 (1.02, 1.07)	0.98 (0.96, 1.01)	1.05 (1.02, 1.08)	1.07 (1.04, 1.10)	1.07 (1.04, 1.10)	1.07 (1.04, 1.10)	1.07 (1.04, 1.10)
% Black	1.25 (1.22, 1.29)	1.17 (1.13, 1.20)	1.26 (1.22, 1.30)	1.29 (1.25, 1.32)	1.29 (1.26, 1.33)	1.29 (1.25, 1.32)	1.29 (1.26, 1.33)
% Hispanic	1.13 (1.08, 1.18)	1.02 (0.98, 1.07)	1.13 (1.08, 1.18)	1.19 (1.14, 1.24)	1.19 (1.14, 1.25)	1.19 (1.15, 1.24)	1.20 (1.15, 1.25)
Population density	1.01 (1.00, 1.02)	1.01 (1.00, 1.02)	1.02 (1.01, 1.03)	1.04 (1.03, 1.05)	1.04 (1.03, 1.05)	1.03 (1.02, 1.04)	1.04 (1.03, 1.05)
Median age	1.07 (1.00, 1.16)	1.01 (0.94, 1.09)	1.05 (0.97, 1.13)	1.04 (0.96, 1.12)	1.03 (0.96, 1.11)	1.06 (0.98, 1.14)	1.04 (0.96, 1.12)
Adjusted R-square	0.4416	0.4818	0.4370	0.4223	0.4202	0.4253	0.4207

All variables were standardized with a mean of zero and a standard deviation of 1. Adjusted Poisson regression controlled for the following zip code level demographics: population density, median age, household income, poverty rate, unemployment, percent with less than a high school education, percent Asian, percent black, percent Hispanic. Log of total population was used as the offset. Zip code coronavirus cases obtained for Arizona, California, Florida, Georgia, Illinois, Maryland, Michigan, Missouri, New York, New Mexico, North Carolina, Ohio, Oklahoma, Pennsylvania, Rhode Island, Texas, Utah, Virginia, Washington, Oregon. *N* = 7625 zip codes.

## Supplementary Materials

The following are available online at https://www.mdpi.com/1660-4601/17/17/6359/s1, Figure S1: Trends of six movement categories using google mobility report data, 15 February to 12 June 2020, United States.

## References

[1] Centers for Disease Control and Prevention (CDC) Cases in the U.S.. https://www.cdc.gov/coronavirus/2019-ncov/cases-updates/cases-in-us.html.

[2] U.S. Bureau of Labor Statistics, the Employment Situation—March 2020; Department Of Labor, United States of America: 2020. https://www.bls.gov/bls/news-release/empsit.htm.

[3] Cajner T., Crane L.D., Decker R.A., Grigsby J., Hamins-Puertolas A., Hurst E., Kurz C., Yildirmaz A. The US Labor Market During the Beginning of the Pandemic Recession; National Bureau of Economic Research: 2020. https://bfi.uchicago.edu/wp-content/uploads/BFI_WP_202058-1.pdf.

[4] Congressional Research Service COVID-19 Pandemic’s Impact on Household Employment and Income. https://crsreports.congress.gov/product/pdf/IN/IN11457.

[5] Garg S. (2020). Hospitalization rates and characteristics of patients hospitalized with laboratory-confirmed coronavirus disease 2019—COVID-NET, 14 States, March 1–30, 2020. Mmwr. Morb. Mortal. Wkly. Rep..

[6] NYC Health Age-Adjusted Rates of Lab Confirmed COVID-19 Nonhospitalizedcases, Estimated Non-Fatal Hospitalized Cases, and Patients Known to Have Died 100,000 by Race/Ethnicity Group as of 16 April 2020. https://www1.nyc.gov/assets/doh/downloads/pdf/imm/covid-19-deaths-race-ethnicity-04162020-1.pdf.

[7] Carrion D., Colicino E., Pedretti N.F., Rush J., Arfer K.B., DeFelice N., Just A.C. (2020). Assessing capacity to social distance and neighborhood-level health disparities during the COVID-19 pandemic. medRxiv.

[8] Bailey Z., Barber S., Robinson W.R., Slaughter-Acey J., Ford C., Sealy-Jefferson S. Racism in the Time of COVID-19. https://iaphs.org/racism-in-the-time-of-covid-19/.

[9] Dyer A. What We Could Learn from the Coronavirus Outbreak on Aircraft Carrier Theodore Roosevelt. Los Angeles Times. 26 April 2020. https://www.latimes.com/california/story/2020-04-26/coronavirus-theodore-roosevelt-aircraft-carrier-outbreak.

[10] Yancy C.W. (2020). COVID-19 and African Americans. JAMA.

[11] Center for Disease Control and Prevention (CDC) How the COVID-19 Spreads. https://www.cdc.gov/coronavirus/2019-ncov/prevent-getting-sick/how-covid-spreads.html.

[12] Hsiang S., Allen D., Annan-Phan S., Bell K., Bolliger I., Chong T., Druckenmiller H., Huang L.Y., Hultgren A., Krasovich E. (2020). The effect of large-scale anti-contagion policies on the COVID-19 pandemic. Nature.

[13] Cheng V.C., Wong S.-C., Chuang V.W., So S.Y., Chen J.H., Sridhar S., To K.K., Chan J.F., Hung I.F., Ho P.-L. (2020). The role of community-wide wearing of face mask for control of coronavirus disease 2019 (COVID-19) epidemic due to SARS-CoV-2. J. Infect..

[14] Urrutia-Pereira M., Mello-da-Silva C., Solé D. (2020). COVID-19 and air pollution: A dangerous association?. Allergol. Immunopathol..

[15] Borck R., Schrauth P. (2019). Population density and urban air quality. CESifo Work. Pap..

[16] Powdthavee N., Oswald A.J. (2020). Is there a link between air pollution and impaired memory? Evidence on 34,000 english citizens. Ecol. Econ..

[17] Pinter-Wollman N., Jelić A., Wells N.M. (2018). The impact of the built environment on health behaviours and disease transmission in social systems. Philos. Trans. R. Soc. B Biol. Sci..

[18] Emeruwa U.N., Ona S., Shaman J.L., Turitz A., Wright J.D., Gyamfi-Bannerman C., Melamed A. (2020). Associations Between Built Environment, Neighborhood Socioeconomic Status, and SARS-CoV-2 Infection Among Pregnant Women in New York City. JAMA.

[19] Keralis J.M., Javanmardi M., Khanna S., Dwivedi P., Huang D., Tasdizen T., Nguyen Q.C. (2020). Health and the built environment in United States cities: Measuring associations using Google Street View-derived indicators of the built environment. BMC Public Health.

[20] Ross C.E., Mirowsky J. (2001). Neighborhood disadvantage, disorder, and health. J. Health Soc. Behav..

[21] Fadini G., Morieri M., Longato E., Avogaro A. (2020). Prevalence and impact of diabetes among people infected with SARS-CoV-2. J. Endocrinol. Investig..

[22] Li B., Yang J., Zhao F., Zhi L., Wang X., Liu L., Bi Z., Zhao Y. (2020). Prevalence and impact of cardiovascular metabolic diseases on COVID-19 in China. Clin. Res. Cardiol..

[23] Sampson R., Raudenbush S. (1999). Systematic Social Observations of Public Spaces: A New Look at Neighborhood Disorder. Am. J. Sociol..

[24] Weaver R. (2016). Evolutionary theory and neighborhood quality: A multilevel selection-inspired approach to studying urban property conditions. Appl. Res. Qual. Life.

[25] Ross C.E., Mirowsky J. (1999). Disorder and Decay: The Concept and Measurement of Perceived Neighborhood Disorder. Urban Aff. Rev..

[26] Burdette A.M., Hill T.D. (2008). An examination of processes linking perceived neighborhood disorder and obesity. Soc. Sci. Med..

[27] Remigio R.V., Zulaika G., Rabello R.S., Bryan J., Sheehan D.M., Galea S., Carvalho M.S., Rundle A., Lovasi G.S. (2019). A Local View of Informal Urban Environments: A Mobile Phone-Based Neighborhood Audit of Street-Level Factors in a Brazilian Informal Community. J. Urban Health.

[28] National Archive of Criminal Justice Project on Human Development in Chicago Neighborhoods. http://www.icpsr.umich.edu/icpsrweb/PHDCN/.

[29] Baltimore Neighborhood Indicators Alliance—The Jacob France Institute Vital Signs 11 Reports. http://bniajfi.org/vs11_report.

[30] de Jong K., Albin M., Skärbäck E., Grahn P., Wadbro J., Merlo J., Björk J. (2011). Area-aggregated assessments of perceived environmental attributes may overcome single-source bias in studies of green environments and health: Results from a cross-sectional survey in southern Sweden. Environ. Health.

[31] Chen J.T., Waterman P.D., Krieger N., Krieger N. (2020). COVID-19 and the unequal surge in mortality rates in Massachusetts, by city/town and ZIP Code measures of poverty, household crowding, race/ethnicity, and racialized economic segregation. Harv. Cent. Popul. Dev. Stud..

[32] Almagro M., Orane-Hutchinson A. (2020). The differential impact of COVID-19 across demographic groups: Evidence from NYC. SSRN.

[33] Almagro M., Orane-Hutchinson A. (2020). The Determinants of the Differential Exposure to COVID-19 in New York City and Their Evolution Over Time. Soc. Sci. Res. Netw..

[34] Chin T., Kahn R., Li R., Chen J.T., Krieger N., Buckee C.O., Balsari S., Kiang M.V. (2020). US county-level characteristics to inform equitable COVID-19 response. medRxiv.

[35] Nesoff E.D., Milam A.J., Barajas C.B., Furr-Holden C.D.M. (2020). Expanding tools for investigating neighborhood indicators of drug use and violence: Validation of the NIfETy for virtual street observation. Prev. Sci..

[36] Simonyan K., Zisserman A. (2014). Very deep convolutional networks for large-scale image recognition. arXiv.

[37] Krizhevsky A., Sutskever I., Hinton G.E. Imagenet classification with deep convolutional neural networks. Proceedings of the Advances in Neural Information Processing Systems.

[38] He K., Zhang X., Ren S., Sun J. Deep Residual Learning for Image Recognition. Proceedings of the IEEE Conference on Computer Vision and Pattern Recognition.

[39] Russakovsky O., Deng J., Su H., Krause J., Satheesh S., Ma S., Huang Z., Karpathy A., Khosla A., Bernstein M. (2015). Imagenet large scale visual recognition challenge. Int. J. Comput. Vis..

[40] Abadi M., Barham P., Chen J., Chen Z., Davis A., Dean J., Devin M., Ghemawat S., Irving G., Isard M. Tensorflow: A system for large-scale machine learning. Proceedings of the 12th {USENIX} Symposium on Operating Systems Design and Implementation ({OSDI} 16).

[41] Paszke A., Gross S., Massa F., Lerer A., Bradbury J., Chanan G., Killeen T., Lin Z., Gimelshein N., Antiga L. PyTorch: An imperative style, high-performance deep learning library. Proceedings of the Advances in Neural Information Processing Systems.

[42] Google 2020-06-12 US Mobility Report. https://www.google.com/covid19/mobility/.

[43] Gibson L., Rush D. (2020). Novel coronavirus in Cape Town informal settlements: Feasibility of using informal dwelling outlines to identify high risk areas for COVID-19 transmission from a social distancing perspective. JMIR Public Health Surveill..

[44] Cyranoski D., Abbott A. (2003). Apartment Complex Holds Clues to Pandemic Potential of SARS.

[45] Seaton A., Godden D., MacNee W., Donaldson K. (1995). Particulate air pollution and acute health effects. Lancet.

[46] To T., Zhu J., Villeneuve P.J., Simatovic J., Feldman L., Gao C., Williams D., Chen H., Weichenthal S., Wall C. (2015). Chronic disease prevalence in women and air pollution—A 30-year longitudinal cohort study. Environ. Int..

[47] Marco M., Gracia E., Martín-Fernández M., López-Quílez A. (2017). Validation of a Google Street View-based neighborhood disorder observational scale. J. Urban Health.

[48] Oliveira E.D.S., Hsu K.-H. (2018). Exploring places of street drug dealing in a downtown area in Brazil: An analysis of the reliability of Google Street View in international criminological research. Int. J. Criminol. Sociol..

[49] Day K., Boarnet M., Alfonzo M., Forsyth A. (2006). The Irvine–Minnesota inventory to measure built environments: Development. Am. J. Prev. Med..

[50] Clifton K., Livi A., Rodriguez D. (2005). Pedestrian Environment Data Scan (PEDS) Tool. Planning.

