# Supplementary material for: Using 164 Million Google Street View Images to Derive Built Environment Predictors of COVID-19 Cases
Source: Int J Environ Res Public Health. 2020 Sep 1;17(17):6359. doi: 10.3390/ijerph17176359 (PMC7504319; doi:10.3390/ijerph17176359)

Supplemental materials for "Using 164 million Google Street View images to derive built environment predictors of COVID-19 cases"

eFigure 1 Trends of six movement categories using google mobility report data, Feb 15 to Jun 12, 2020, United States.

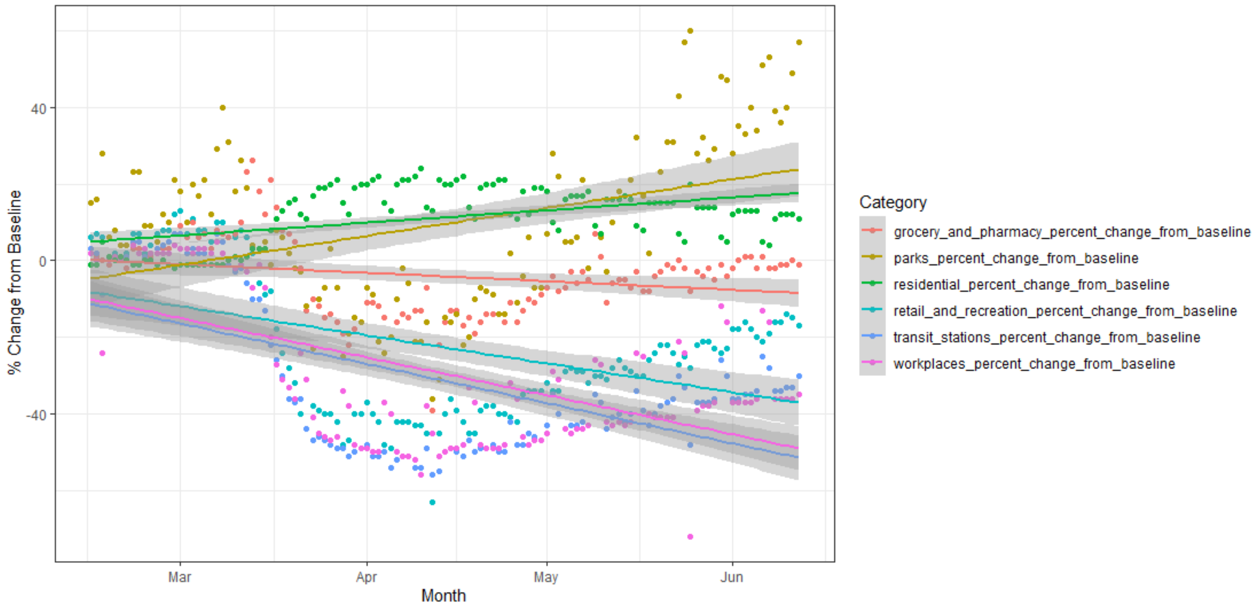

Supplement: Supplementary file 1 [file ijerph-17-06359-s001.pdf]
